# Aspiring physicians program: description and characterization of the support processes for an undergraduate pathway program to medicine

**DOI:** 10.1080/10872981.2023.2178368

**Published:** 2023-02-15

**Authors:** Arianne Teherani, Kelechi Uwaezuoke, Jazmine Kenny, Connie Calderón-Jensen, Tomás Magana, Katherine Flores, Alicia Fernandez

**Affiliations:** a School of Medicine, Center for Faculty Educators, University of California, San Francisco, California, USA; b Inclusion and Community Partnerships at SF BUILD, San Francisco State University, San Francisco, CA, USA; c University of California, Merced, Merced, CA and Latinx Center of Excellence, University of California, San Francisco, School of Medicine, San Francisco, CA, USA; d School of Medicine, Latinx Center of Excellence, University of California, San Francisco, California, USA

**Keywords:** Pathway programs, undergraduate, diversity, evaluation, underrepresented in medicine

## Abstract

Latinx physician rates are lower than non-Latinx white physicians. Many pathway programs to careers in medicine have been established for underrepresented students, yet few focus on premedical college education or undergraduate pathway programs, which marks a critical junction in the commitment to and preparation for application to medical school. Moreover, little is known about the program components which prepare and support learners. Framed by Swail’s Model for Persistence and Achievement, we characterize how a given program’s components impact support and growth for participating students. Using the process step of the Context, Input, Process, and Product evaluation model, we conducted focus groups at the end of the program, with four cohorts of student participants between 2019 and 2022. Focus groups identified strengths and limitations in content and delivery to improve program effectiveness and plan for the future of a program. We used thematic analysis, following an inductive approach, to analyze data from transcribed focus groups. A total of 66 of 81 (81.5%) students participated in focus groups. Students described that supportive program components include long-term mentorship and advising that builds trust, academic preparation for medical school, early exposure to clinical career exploration, tools to articulate students’ personal narrative, methods to recognize and address challenging situations in the professional environment, community leadership development, and leveraging health policy and advocacy to empower students to create systems change within communities. Our findings affirm and provide a needed account of program components known to be contributors to student success in undergraduate pathway programs. Our evaluation also characterizes additional supportive processes not discussed elsewhere. Our findings contribute to knowledge about development and implementation of undergraduate pathway programs and the components by which these programs create opportunities for success among underrepresented students aspiring to careers in medicine.

Latinx physician rates in the United States are at 6% and in California are nearly 90% lower than non-Hispanic white physicians [1,2]. In 2017, California medical schools graduated 110 Latinx medical doctors, a rate at which it would take almost 500 years until the Latinx physician rate is equitable to non-Hispanic white physicians [1]. Studies examining this disparity have identified several contributing factors to underrepresentation in medicine (UIM) including ‘low-performing high schools’ not preparing UIM students for the rigors of the pre-medicine track in college and the process to apply to medical school, financial cost of medical education, limited educational and advising resources, Medical College Admissions test (MCAT) scores, lack of access to UIM role models in medicine, and sociocultural barriers, such as stereotype threat [3–7]. Many pathway (also referred to as pipeline) programs for careers in medicine have been established to create equitable opportunities and preparations to apply to medical schools ranging from middle and high school, college, and post-baccalaureate and range in duration from single-day sessions to multi-year programs. Although much has been described about these wide-ranging programs and their outcomes, most focus on the post-baccalaureate track [8–10] and few describe the premedical undergraduate education track known as undergraduate pathway programs, when students are still in college, which marks a critical junction in the commitment to and preparation for application to medical school [11]. Limited descriptions of such programs show that they promote diversity in medical education through a primary focus on academic support [12–16]. Little is known about the structure and process by which these programs prepare learners academically and otherwise.

Swail’s [17] Model for Persistence and Achievement asserts that student persistence and achievement are the results of interaction between the cognitive (e.g., academic preparation and support), social (i.e., peer and familial encouragement, sociocultural barriers), and institutional (i.e., institutional practice including academic and social support, and curriculum) factors. The ability of the educational institutions to leverage these factors to promote student success is critical. To optimize success, pathway programs must consider all factors that influence student experiences and interactions within their education environment as well as their future practice.

Existing undergraduate pathway program descriptions focus primarily on cognitive factors, such as academic preparation and include limited evaluation outcomes. These programs include offerings on foundational science coursework [13,15,16,18,19], learning support strategies [6,13,15,18–20], research [14,21], exposure to clinical settings [15], guidance on how to apply to medical school including preparation for taking the MCAT and financial aid [6,7,14,20], use of mentors and advisors [7], and opportunities for social activities [15,21]. Yet, social factors beyond academic achievement impact UIM learner’s choice to select a career in medicine including identity, awareness of and skill with which to confront sociocultural barriers (e.g., experiences with educational inequities and bias), and preparation to address societal health concerns and health-care disparities. A limited number of undergraduate pathway programs describe preparing UIM learners academically, in addition to addressing societal health concerns, professional socialization, and identity [20,21]. Moreover, evaluation of undergraduate pathway programs has been primarily anecdotal [14,16,19] and, when available, limited in focus to program satisfaction [20,21], preparation for successful application and admission to medical school [11,16,18–20], and medical school performance outcomes [15]. Few program descriptions explore and characterize the undergraduate pathway program components which cultivate support. Understanding these processes is particularly important as some undergraduate pathway programs have had greater success with medical school preparation compared to others [11,22].

We describe an undergraduate pathway program designed to address the cognitive, social, and institutional factors that prompt achievement and characterize the way the program components support participating students. Our work has implications for how undergraduate pathway programs create opportunities for success among UIM students aspiring to careers in medicine.

## Materials and methods

### Context

With funding from the Health Resources and Services Administration (HRSA) and the University of California, San Francisco, School of Medicine (UCSF SOM), the Aspiring Physicians Program (APP) is a medical preparatory program for underrepresented college students. The UCSF SOM is a public, research-intensive institution in the western United States that graduates approximately 165 medical students annually. The APP was created by the UCSF SOM Latinx Center of Excellence, in collaboration with two public regional state universities: California State University, San Francisco (SFSU) and California State University, Fresno (Fresno State). SFSU and Fresno State are part of the California State University System made up of 23 campuses. A vast majority of California State University graduates remain in California post-graduation. SFSU enrolls approximately 27,000 and Fresno State 25,000 students annually. SFSU is a public urban university, ranked in the top 25 of the Social Mobility Index [23]. Fresno State serves the diverse rural region of central California and is designated by the US Department of Education as both a Hispanic-Serving Institution and an Asian American and Native American Pacific Islander-Serving Institution [24]. For the last three years, a few students from University of California Merced (UCM) joined the Fresno State cohort. UCM is part of the 10 campus University of California system. Located in central California, it enrolls approximately 8000 students annually with 75% of students being first-generation to college [25].

### Design and sample

The primary focus of our evaluation was to characterize how the APP components contributed to student support and growth. Hence, we drew on the *process* step of the Context, Input, Process, and Product (CIPP) evaluation model. The model leverages data collection to identify strengths and limitations in content or delivery, to improve program effectiveness or plan for the future of a program [26,27]. A total of four cohorts of 81 students participated in APP between 2019 and 2022. The UCSF Institutional Review Board approved the study.

### Program overview

APP begins as a 6-week intensive summer program that supports pre-medical junior and senior underrepresented students who have completed their biology and chemistry classes with an interest in pursuing medicine careers. Students from backgrounds historically underrepresented in medicine, often have an interest and not the knowledge nor support needed to navigate the medical school application process [28]. Hence, our evaluation sought to explore and characterize how program components contribute to student support in such programs. Latinx students within the program are supported through a summer stipend provided by the Latinx Center of Excellence while other underrepresented students are supported by the UCSF Office of Diversity and Outreach funds. In 2019, students attended APP in person while in 2020 and 2021, students attended virtually, due to COVID-19 restrictions. When COVID restrictions lifted in 2022, the program was delivered in a hybrid model. APP is built on three core pillars, all of which integrate at least one of Swail’s [17] factors: (a) medical school preparation (social, cognitive, and institutional), (b) personal and professional development (social), (c) health policy and advocacy (social, cognitive, and institutional). Table 1 displays and describes each pillar, the components within that pillar, and where relevant, the literature and evidence supporting the design and/or implementation of each component. In addition to the pillar and its components, the program was followed by ongoing continuity in support. Building on the importance of continuity in experience, post-culmination of the 6-week summer component of APP, students receive year-long longitudinal academic support. The program provides individualized, student-centered mentorship and guidance longitudinally that builds upon the fundamentals taught during the summer course [29]. In preparation for application to medical school, students collaborate with their APP faculty mentors on preparing their application, refining their personal statements, obtaining letters of recommendation, and submitting their application early. Students who chose to temporarily delay applying to medical school, work with faculty mentors to define their post-graduation plans, including support with post-baccalaureate program applications, identifying employment and training opportunities, connections to community networks, and establishing a timeline for medical school application.

**Table 1.** APP pillars, components within that pillar, description of each component, and the literature and evidence utilized in the design and/or implementation of each component. UCSF.

Component of Pillar	Description of Component	Literature and Evidence Utilized to Design and/or Implement Component (If Relevant)
Program Pillar: Medical School Preparation		
*Medical school readiness*	This component builds students’ knowledge about the medical school application process, provides information about resources and on creating a competitive application, and mock interviews with feedback.	Hadinger MA. Underrepresented Minorities in Medical School Admissions: A Qualitative Study. *Teach Learn Med*. 2017;29(1):31–41.
*Structure of medical training*	APP educates students about the format of medical training starting with pre-clinical years and into residency with the aim of preparing students for what to expect and how to work through the implicit rules of medical education to succeed.	Billett S. *Learning in the Workplace: Strategies for Effective Practice*. Allen and Unwin; 200127.Hafferty FW. Academic Medicine and Medical an Evolving Field of Educational Scholarship. *Academic Medicine*. 2018;93(4):532–536.
*Preparation for the MCAT*	Building on evidence supporting the effectiveness of near peer teaching, MCAT preparation takes place with near-peer facilitators (students with teaching experience and successful MCAT performance).	Chou CL, Teherani A. A foundation for vital academic and social support in Clerkships: Learning through peer continuity. *Academic Medicine*. 2017;92(7).
*Career exploration*	Students learn about varied career choices by engaging with guest speakers, including introduction to academic medicine.	
*Customizing a personal pathway to medical school*	APP informs students about the multiple pathways into medical school often informed by personal choices, financial and psychosocial constraints, family needs, and other cultural factors.	
*Program Pillar: Personal and Professional Development*		
*Articulation of the personal narrative*	These sessions increase students’ abilities to articulate (for the medical school application) their lived experiences and contributions through an asset-based lens.	O’Connor C. 2019 Wallace Foundation Distinguished Lecture: Education Research and the Disruption of Racialized Distortions: Establishing a Wide-Angle View. *Educational Researcher*. 2020;49(7):470–481
*Underscoring the importance of mentorship*	Considering decades-long research showing the importance of mentorship to UIM student success, APP addresses what a successful mentoring relationship is and how to engage mentors. Students are matched with a program mentor.	Farkas AH, Allenbaugh J, Bonifacino E, Turner R, Corbelli JA. Mentorship of US Medical Students: a Systematic Review. *J Gen Intern Med*. 2019;34(11):2602–2609 Harris TM, Lee CN. Advocate-mentoring: a communicative response to diversity in higher education. *Commun Educ*. 2019;68(1):103–113
*Recognition of sociocultural barriers in the professional environment*	These sessions, grounded in the evidence on UIM learner experiences, equip students with the tools necessary to identify structural and non-structural barriers to success and provide tools for overcoming barriers. These sessions address topics such as overcoming stereotype threat, imposter syndrome, and unconscious bias, and working through microaggressions.	Odom KL, Roberts LM, Johnson RL, Cooper LA. Exploring Obstacles to and Opportunities for Professional Success Among Ethnic Minority Medical Students. *Academic Medicine*. 2007;82(2):146–153 Orom H, Semalulu T, Underwood W. The social and learning environments experienced by underrepresented minority medical students: A narrative review. *Academic Medicine*. 2013;88(11):1765–1777 Bullock JL, Lockspeiser TM, del Pino-Jones A, Richards R, Teherani A, Hauer KE. They don’t see a lot of people my color: a mixed-methods study of racial/ethnic stereotype threat among medical students on core clerkships. *Academic Medicine*. 2020; 95 (11), S58–66. Sukhera J, Gonzalez C, Watling CJ. Implicit Bias in Health Professions:From Recognition to Transformation. *Academic Medicine*. 2020;95(5):717–723
*Importance of self-care*	In response to heightened awareness of physician burnout APP prepares students to meet the challenges of future medical education and provide them with effective stress management tools.	Thomas LR, Ripp JA, West CP. Charter on Physician well-being. *JAMA – Journal of the American Medical Association*. 2018;319(15):1541–1542.
*Leadership Development*	As leadership training is key to the success of underrepresented learners, a key component of APP is to empower students to see themselves as leaders within their communities.	Leblanc C, Sonnenberg LK, King S, Busari J. Medical education leadership: from diversity to inclusivity. GMS J Med Educ. 2020; 37(2):Doc18
*Program Pillar: Health Policy and Advocacy*		
*Fundamentals of public health and health policy*	Students are introduced to foundational public health concepts including health disparities, social determinants of health, population health data measurements, and health disparities.	Dawes DE. Perspective: The future of health equity in America: Addressing the multiple, intersecting determinants of health. *Ethn Dis*. 2019; 29:343–344
*Policy advocacy*	Sessions provide an overview of health policy development, data collection and needs assessments, policy communication, and advocacy. Adapted from an existing learning approach students engage with a writing activity and mock debate on the topic of universal healthcare.	Hubinette M, Dobson S, Scott I, Sherbino J. Health advocacy*. *Med Teach*. 2017;39(2):128–135 Payán DD. Cultivating Health Policy Analysis and Communication Skills in Undergraduate Public Health Education: An Active Learning Approach. *Pedagogy Health Promot*. 2021;7(3):235–241
*Policy fact sheets*	Students learn about community engagement, meet with community-based organizations (CBOs), and learn how to create a policy fact sheet using the key principles for policy fact sheet development. To date, more than half of CBOs report using these factsheets for their organizations.	American Public Health Association. Fact Sheets. American Public Health Association
*Empowerment and community advocacy*	Students are taught to recognize their role as community advocates and envision ways to make a difference even prior to starting their careers as physicians.	

### Instrument

We used focus groups to explore and characterize the processes by which the program created support and success for participating students. The focus group guide, displayed in Table 2, was created by three investigators (AT, TM, and AF). The guide asked about program perception and the various components within each of the APP pillars. All students were invited to participate in the focus groups; participation was voluntary. An investigator (AT) conducted the focus groups.

**Table 2.** Student participant focus group guide utilized for data collection about the program components of the APP between 2019 and 2022.

Focus Group Questions and Probes
To start us off, briefly, reflect back on your experiences during the **APP**. Tell us more about your overall experience.
(2) Could you describe the key lessons you learned about health professions careers/medicine during your time in **APP**? About other careers? Any other key lessons learned during your time in **APP**?
(3) Did the program make you aware of how to become a successful pre-medical student and medical student applicant? Please elaborate.
(4) How would you describe your interest in medicine prior to participating in the program? (a) Probe: How would you describe your interest in health professions/medicine after participating in the program?
(5) How would you describe your understanding and interest in health policy and working with communities prior to participating in the **APP**? (a) Probe: Did participation in the program impact your interest in health policy? Working with communities? If so, please describe how? How do you see being involved health policy efforts moving forward? (b) Probe: In the future, will you engage in health policy efforts relating to the topic/in the area you worked on during the program? Communities? Why or why not?
(6) Let’s focus now on the activities (e.g., workshops, mentoring, project work, group meeting, fact sheet development, provided to you as part of program. Overall, how valuable were those activities to your learning? You may discuss individual activities or activities a whole. (a) Probe: Were there activities that were more or less helpful and why?
(7) How would you describe your interest in a career in medicine after participating in the program? (a) Probe: Did participation in the program impact your interest in medicine? If so, how? Please describe. (b) Probe: Which program activities do you think were most important in impacting your career decisions?
(8) Is there anything about the program that we have not asked about that you think is important to know?

### Data analysis

We used thematic analysis [30] following an inductive approach, to analyze data from transcribed focus groups. We selected the inductive approach because it is built on the principles of the constructivist paradigm which focuses on how individuals understand and create their own meaning from life events/education. This was fundamental to the purpose of our evaluation study, which was meant to characterize the way program components support participating students. Moreover, as is traditional for qualitative methods, we sought to understand program components that may be transferable to other settings and establish next steps in the study of undergraduate pathway programs. One author (AT) and a research assistant developed a codebook based on the first focus group transcription with preliminary, low-inference codes (observable data expressed in a non-judgmental way) which was further refined through an iterative approach with each of the subsequent focus group transcripts. Since our aim was to characterize how program components support participating students, we focused primarily on analyzing student perceptions of program components. During this latter step, the codebook was refined through ongoing analysis of additional transcripts, and newly added/revised codes were iteratively re-applied to prior transcripts. Each transcript was coded by one author (AT) and a research assistant and reconciled through discussion. All authors reviewed overarching themes synthesized based on the analyzed data each year. We organized coded transcripts with Dedoose Analytic Software (SocioCultural Research Consultants, LLC, Manhattan Beach, California).

### Reflexivity

The program development and study team included one man and six women (including a research assistant who helped analyze the data) of diverse professional roles; the study team consisted of physicians, education experts, a research assistant, and education and public health researchers. Most investigators identified as Latinx, 1 as Black, 1 as biracial, 1 as Southwest Asian, and 1 as White.

## Results

Demographics for all 81 student participants are displayed in Table 3. We conducted four focus groups, at the end of each summer program, with a total of 66 of 81 (81.5%) participants (11 in 2019, 18 in 2020, 17 in 2021, 20 in 2022). Of all the focus group participants, 47 of the 66 participants identified as women (71.2%) and 19 (28.8%) men. Fifty-eight (87.9%) were from Latinx, 5 (7.6%) were African American, and 3 (4.5%) were biracial or from other historically underrepresented groups. The focus groups lasted between 46 and 62 min.

**Table 3.** Demographic characteristics of student participants in the APP at the UCSF between 2019 and 2022 (*N* = 81).

	APP *N*=81									
	Race/ethnicity				Gender			Undergraduate Institution		
Year	Latinx *N* (%)	African American *N* (%)	Bi-Racial *N* (%)	Other UIM *N* (%)	Female *N* (%)	Male *N* (%)	Nonbinary *N* (%)	California State University, Fresno *N* (%)	California State University, San Francisco *N* (%)	University of California, Merced *N* (%)
2022	16 (19.8)	2 (2.5)	2 (2.5)	1 (1.2)	15 (18.5)	6 (7.4)	0	10 (12.4)	10 (12.4)	1 (1.2)
2021	18 (22.2)	1 (1.2)	1^a^ (1.2)	0	16 (19.8)	4 (5)	0	8 (9.9)	10 (12.4)	2 (2.5)
2020	18 (22.2)	0	0	2 (2.5)	14 (17.3)	5 (6.2)	1 (1.2)	9 (11.1)	10 (12.4)	1 (1.2)
2019	16 (19.8)	2 (2.5)	0	2 (2.5)	14 (17.3)	6 (7.4)	0	9 (11.1)	11 (13.5)	0
All years	68 (84)	5 (6.2)	3 (3.7)	5 (6.2)	59 (72.8)	21 (25.9)	1 (1.2)	36 (44.4)	41 (50.6)	4 (5)

^a^Latinx and African American.

Students described how the program helped them gain confidence, increased their enthusiasm, and affirmed their ambitions to enter medicine. The program provided students with valuable information about the pathway to, through, and beyond medical school.

*“I was scared because I’m an immigrant and having language barrier … I was thinking there’s a very great chance that I wouldn’t get into medical school … But with this program I believe now that I can do it. So it’s just great…it inspired me a lot that I can do it now.” (R10, 2020)*

Students also realized the importance of not rushing, but to progress at their own pace to apply to medical school and to stay committed throughout and beyond because learning is an ongoing activity.

*“ … now I have a clear picture of what to do. Because before, I just didn’t know how it was going to happen, and now I have the prep year. Before everybody was like, ‘Just go because it’s going to take forever. You might as well just go quickly and just go through it so you can continue your profession already,’ but you need to take breaks. It’s inevitable, and you don’t want to get burnt out. The prep year is definitely something I think I’m going to consider.” (R12, 2022)*

### Characterization of supportive program components

Students described supportive components of the program in a way that reflected the existing structure of the program pillars. We describe key themes and Table 4 displays representative quotes for themes described.

**Table 4.** Themes and quotes by theme characterizing supportive processes of the APP pillars described by participating students in four focus groups between 2019 through 2022.

Theme	Quote (participant focus group year)
Medical School Preparation	The MCAT prep was the bread and butter for me. As an aspiring physician … going to medical school, there is a standardized test that you have to do well on in order to get in. That was … the highlight of the program … Because … if you don’t … get how to do it, then that cannot serve you best” (2019) We should do more MCAT prep exercises because those are really hard and you need to practice … instead of just an hour, maybe two … we need to practice and practice for the MCAT. So … more time for that will be better. (2021) I would definitely love to see more clinical skills demonstrated and practiced … and … being able to possibly check out some … clinics … that we are affiliated with … would be a welcome addition to the program.” (2019) The program should implement more … problem-based learning because … that was very helpful … on how to help future patients as well as more like suturing or like more hands-on activities like anatomy, physiology because we learn more from those activities I believe as future doctors.” (2021) Something that I really enjoy about the program is that it … reemphasizes the idea that you could be a physician but … you can also be a policy maker … an educator. You can wear different hats … that’s something … I really enjoyed hearing and learning about because … to revolutionize healthcare, we do have to take a different approach … So, the program reenergizing the idea that that’s possible was really refreshing.” (2019) Knowing … what else I can do. Hearing about I can get a Masters. I could take a stop in med school or follow some other degree I want … It definitely changed my perspective [of] … you just have to solely be in medicine … there’s … other aspects you could pick up … like a post-bac and stuff like that to solidify.” (2021) I know the focus was physicians, but there was a sprinkle of just different career opportunities; PhD research and health policy and things like that within healthcare.(2022)
Personal and Professional Development	… had a lot of self-reflecting … There were a lot of exercises that … had us look back on our experiences or on ourselves as a person … and that was … very helpful to understand who you are and build yourself up from there. (2019) My experience … has been really positive overall. I feel like we worked a lot on personal development and being able to tell our story and represent ourselves, our identity when we’re applying for medical school, and have that safe space to be able to talk to the different teachers and mentors about our story. (2020) How helpful and impactful it is for me to have been able to interact with some Black doctors because … I’ve never had a Black doctor until I was 19 and I’m 20 now and I’ve only met one Black nurse and that’s my dad. So, like having the experience to speak with Black physicians and Black med students was so emotional for me, but also so powerful for me as well. (2021) Overall … the sense of community … in this program … it’s good to be surrounded by people of color that are also on the same path. It’s like, ‘Okay, we can all do this.’ We all uplifted each other. (2019) I hope that the program can continue to have more interactions between the San Francisco cohort and the Fresno cohort, because this is really great to be able to get to open up the experience toward a lot of people and get to hear other perspectives in other communities. (2020) I found very valuable the networking … I didn’t know that there’s so many people that want to help us into our journey into medical school. (2021) There’s definitely an emphasis on finding and building your networks. Every physician we talked to in the program emphasized how important it is to have those connections and networks, especially those of us who are first generation whose parents aren’t necessarily very affluent. We need those to get off the ground. (2022) Mentorship … that one hit really deep because before this I was … going through the motions by yourself and trying to figure it out. Because … I don’t really trust other people to help me but now I reach out to … people … [to] mentor me … because they know that we’re going to make an impact or we’re what’s needed in the future … So, it was really good. (2021) The microaggression, micro-affirmation block … was awesome. I had no real knowledge of that topic, let alone the ramifications that come with it … it kind of illuminated a lot of … examples in my own life that…I didn’t know what to call … [Dr T] gave perfect examples for us to follow … that to this day, I’m still moving forward with. I loved that. (2019)
Health Policy and Advocacy	We’re doing policy … I had no idea that that was going to be the focus … So, this program is really good in showing us how physicians have a voice that can reach congress, or the president, or our local board of supervisors. Especially as undergrads, we have a lot of power given the fact that this is an opportunity where we can reach up to those people. Those would be your congressmen, senators, whatever. (2019) I definitely want to make pushes to better help the community with a lot of the policies that physicians and people with that kind of status, power, wealth are able to make just in a day because it’s important. I feel like, especially for the XX community in a lot of the XX, the underserved community, definitely want to push for better healthcare access and options for everybody. I think I definitely want to pursue that in the future. (2022) Before [the program] I thought it was strictly like just medicine, you’re a doctor, that’s it … I’ve learned now that it’s medicine and also with advocacy, and really needing to know all the health policies, and how you play a role in changing them. (2020) Overall **…** I … took away that the health policy does intertwine with medicine. I thought they were completely separate … and … now … I get it … [and] understand why they are together and why people speak from public health officials. This course taught me that and I’m very grateful for that. (2021) The talk … today … was helpful to our policies, but … began at the government level and … state level where … I don’t think we would have been able to get our policy all the way up there. So … maybe implementing it … at the local level, community-wise where … we’re actually doing with our policies even at just a local level, and having … the same information … but in a … way that applies to what we can do and what we have the power to do. (2019) The content was a little … broad or general … trying to cover everything on surface level … I would rather we had focused in on a certain area that maybe pertain to what we could do as doctors or … with students or … learning how to create a policy ourselves … might have been … more useful for me because … I enjoyed going to class and the conversations but I didn’t feel really engaged in it. (2021) It was very broad … trying to teach … everything that goes on into making a policy and a bill, and … it would have been nice to have it centered on one specific aspect of that. (2021) A lot of [students] struggled with their CBO’s … if the CBO’s had a better understanding … those sessions would have gone a lot better. (2021) if there was maybe something we could do … to say, “These are certain physicians and some subspecialties or fields that are affiliated with our schools in your areas that are willing or want a medical translator for English, Spanish, Hmong, Punjabi or they’re looking for someone to actively shadow or to actively mentor under (2022)

#### Medical school preparation

Students realized the value of having a clear vision of medical school application process and the vital role of the MCAT. They appreciated the preparation provided by the program. Suggestions for increasing the impact of preparation sessions included increased time dedicated to MCAT preparation and additional feedback on writing activities.

Many students would have liked more practical and clinical activities, such as problem-based learning cases and more opportunities to be in a clinical environment. The program also shifted students’ perceptions about medicine and health professions careers by presenting opportunities they had not considered or been aware of as possibilities previously.

#### Personal and professional development

The program provided students an important space to self-reflect and be open about their identity. Students were inspired by program participants (e.g., medical students, and faculty) with similar backgrounds who, despite feeling disadvantaged, overcame similar difficulties. This helped students realize their potential as competitive applicants and that medical school admission was possible.

Throughout the program, there were opportunities for community building across different campuses. Students appreciated time set aside to engage, learn, and network with other students and program faculty with similar backgrounds. Students highlighted the importance of learning how to communicate with faculty when working with them on medical school interviews.

Students reported how APP mentorship taught them to trust and seek help from others. Students underscored the importance of seeking out mentors in the future. Students also highlighted the value of learning about moving beyond sociocultural barriers such as microaggressions and stereotype threat, to achieve their educational goals.

#### Health policy and advocacy

Students appreciated learning about health policy and advocacy, and some expressed interest in pursuing these areas further. Some students recommended more time be dedicated to health policy and advocacy, including fact sheet development. This included early communication about and emphasis on the value of health policy and advocacy activities so that students could make connections and apply what they learned in preparation for medical school. Moreover, students appreciated working directly with and learning from local CBOs and helping with locally identified needs. Students also noted that CBOs having a full understanding of the program and its purpose enhances ongoing communications and partnerships.

## Discussion

Focused on three core pillars of medical school readiness, personal and professional development, and health policy and advocacy, we describe an undergraduate pathway program and its components aimed at supporting underrepresented college students in their application to medical school. Our findings reaffirm and provide needed account of program components known to be contributors to student success in undergraduate pathway programs. These include the value of mentorship and advising that is longitudinal and builds trust [7], importance and format of academic preparation for medical school including the MCAT [6,14,20], and desire for early exposure to clinical setting [15]. Our evaluation also characterized additional supportive processes not discussed elsewhere. These included the value of career exploration and knowledge about the multiple pathways to medical school, tools to articulate students’ personal narrative, methods to recognize and address sociocultural barriers in the professional environment, ways to enable students to be community leaders, and leveraging an education in health policy and advocacy to empower students to understand and create systems change within communities. These findings together contribute to the knowledge about development and implementation of undergraduate pathway programs. The program components we identified in this study form the foundation of student success in undergraduate pathway programs. Next steps must explore these components further within existing and future programs and varied settings to determine broad applicability and transferability.

With an aim to empower students to understand and create systems change to address social determinants of health, the third pillar of the APP program addressed health policy and advocacy [31,32]. Advocacy is considered a value of the medical profession with many organizations in the United States (US) acknowledging its important role in the profession [32]. Yet, advocacy is not a core competency for US medical graduates and is not taught consistently across core curriculum similar to other countries that follow the CanMeds framework which include the health advocate role as one of the seven core physician roles [33]. The APP program commences advocacy education prior to medical school, instilling value and providing students with the needed framework and tools to enact change in the community and for patients. Moreover, some APP students expressed interest in pursuing this work into future practice. Future research should address the long-term learning and transfer of advocacy skills into medical schools and beyond from programs like APP.

A considerable amount of work has identified the lack of underrepresented role models in premedical training as a key barrier to underrepresented student application into medical school [7,34]. Likewise, we found that APP students appreciated the opportunity to be around those with similar backgrounds, learn from underrepresented faculty and medical students while building communities and receiving ongoing mentorship as key components that positioned them for success. Our findings resonate with Uwaezuoke’s [34] evidence-based recommendations to address environmental and contextual barriers reported, which focus on creating college-based opportunities for underrepresented premedical learner communities, more underrepresented instructors throughout the continuum, and opportunities for students to interact with faculty in informal settings [34]. Aligned with these recommendations, our findings echo needed and established components that all undergraduate pathway programs should include to promote student success.

We drew on Swail’s [17] Model for Persistence and Achievement which emphasized the interaction between the cognitive, social, and institutional factors. Recent work has proposed expanding the model to place students at the center and explores the institution’s role in shaping students’ experiences while accounting for individual- and interpersonal-level factors [34]. Individual factors include academic abilities and strengths and weaknesses of the student. Interpersonal factors include personal attitudes, cultural background, and social interactions with others in the education setting (e.g., peers and faculty). The APP program itself was an institutional factor that provided opportunities for growth to students both on the individual and interpersonal level aimed to intentionally support student persistence and achievement in applying to medical school. Yet underrepresented student persistence and resiliency are vital to this success and must be accounted for. For instance, it is known that underrepresented premedical students encounter negative advising experiences [7] which create mistrust and deters them from applying to medical school, and yet many students persist, and programs can contribute to positive persistence and ultimate success [35]. Ultimately, to support student persistence and achievement, cohesive and successful endeavors require attention to all aspects, be it cognitive, social, individual, interpersonal, and institutional.

Limitations to our program and evaluated outcomes included context and timing. Our program was developed in a state and within a public university system with a diverse demographic that may not be similar to other institutions nationwide. More research is needed to explore whether our findings are transferable, under which conditions, to other contexts. We did not include students’ long-term outcomes for success into entering and persisting through medical school. We are currently following our student through medical school application.

## Conclusion

We describe an undergraduate pathway program designed to address cognitive, social, and institutional factors built on three core pillars of medical school preparation, personal and professional development, and health policy/advocacy. We characterize program components that support participating students. These supportive processes include long-term mentorship and advising that builds trust, academic preparation for medical school, early exposure to clinical career exploration, tools to articulate students’ personal narrative, methods to recognize and address sociocultural barriers in the professional environment, community leadership development, and leveraging health policy and advocacy to empower students to create systems change within communities. Our findings contribute to knowledge about the components by which undergraduate pathway programs create opportunities for success among UIM students aspiring to careers in medicine.
